# Novel Root-Fungus Symbiosis in Ericaceae: Sheathed Ericoid Mycorrhiza Formed by a Hitherto Undescribed Basidiomycete with Affinities to Trechisporales

**DOI:** 10.1371/journal.pone.0039524

**Published:** 2012-06-25

**Authors:** Martin Vohník, Jesse J. Sadowsky, Petr Kohout, Zuzana Lhotáková, Rolf Nestby, Miroslav Kolařík

**Affiliations:** 1 Department of Mycorrhizal Symbioses, Institute of Botany, Academy of Sciences of the Czech Republic, Průhonice, Czech Republic; 2 Department of Experimental Plant Biology, Faculty of Science, Charles University in Prague, Prague, Czech Republic; 3 Bioforsk Grassland and Landscape Division, Norwegian Institute for Agricultural and Environmental Research, Stjørdal, Norway; 4 Laboratory of Genetics, Physiology and Bioengineering of Fungi, Institute of Microbiology, Academy of Sciences of the Czech Republic, Prague, Czech Republic; Jyväskylä University, Finland

## Abstract

Ericaceae (the heath family) are widely distributed calcifuges inhabiting soils with inherently poor nutrient status. Ericaceae overcome nutrient limitation through symbiosis with ericoid mycorrhizal (ErM) fungi that mobilize nutrients complexed in recalcitrant organic matter. At present, recognized ErM fungi include a narrow taxonomic range within the Ascomycota, and the Sebacinales, basal Hymenomycetes with unclamped hyphae and imperforate parenthesomes. Here we describe a novel type of basidiomycetous ErM symbiosis, termed ‘sheathed ericoid mycorrhiza’, discovered in two habitats in mid-Norway as a co-dominant mycorrhizal symbiosis in *Vaccinium* spp. The basidiomycete forming sheathed ErM possesses clamped hyphae with perforate parenthesomes, produces 1- to 3-layer sheaths around terminal parts of hair roots and colonizes their rhizodermis intracellularly forming hyphal coils typical for ErM symbiosis. Two basidiomycetous isolates were obtained from sheathed ErM and molecular and phylogenetic tools were used to determine their identity; they were also examined for the ability to form sheathed ErM and lignocellulolytic potential. Surprisingly, ITS rDNA of both conspecific isolates failed to amplify with the most commonly used primer pairs, including ITS1 and ITS1F + ITS4. Phylogenetic analysis of nuclear LSU, SSU and 5.8S rDNA indicates that the basidiomycete occupies a long branch residing in the proximity of Trechisporales and Hymenochaetales, but lacks a clear sequence relationship (>90% similarity) to fungi currently placed in these orders. The basidiomycete formed the characteristic sheathed ErM symbiosis and enhanced growth of *Vaccinium* spp. *in vitro*, and degraded a recalcitrant aromatic substrate that was left unaltered by common ErM ascomycetes. Our findings provide coherent evidence that this hitherto undescribed basidiomycete forms a morphologically distinct ErM symbiosis that may occur at significant levels under natural conditions, yet remain undetected when subject to amplification by ‘universal’ primers. The lignocellulolytic assay suggests the basidiomycete may confer host adaptations distinct from those provisioned by the so far investigated ascomycetous ErM fungi.

## Introduction

Ericaceae comprise approx. 3000 species accommodated in 100 genera which are native to all continents except Antarctica. Generally, they are calcifuges ubiquitous in acidic soils with high organic content, often dominating ecological niches that they inhabit. Ericaceae display great morphological and ecological plasticity, including such different forms as Himalayan tree dominants, inconspicuous epiphytic species inhabiting tropical forests, or resilient subarctic dwarf shrubs. They also include potentially invasive species, e.g., *Gaultheria shallon* in western coast of Canada, valued ornamentals, e.g., the genus *Rhododendron*, and commercially important crops, e.g., cultivated highbush blueberry, *Vaccinium corymbosum*.

Ericaceae depend strongly on mycorrhizal fungi for nutrient acquisition from soil organic matter. Arbutoid/pyroloid and monotropoid ectendomycorrhizae and ericoid endomycorrhizae are well-described and morphologically distinct types of mycorrhizae in Ericaceae [1]. The ericoid clade of Ericaceae [2] ( =  Ericaceae in the following text) primarily hosts ericoid mycorrhizal (ErM) symbiosis, a specialized type of endomycorrhiza in which dense hyphal coils are contained in rhizodermal or cortical cells of fine (∼100 µm diam.) hair roots with a simple anatomy and ephemeral lifespan [3]. Extraradical mycelia of ErM fungi do not reach more than a few mm from the root surface into the surrounding soil [4] and, unlike ectomycorrhizae, ericoid mycorrhizae typically lack multilayered hyphal mantles, with exceptions including the superficial hyphal sheaths reported on *Gaultheria procumbens* by Massicotte *et al*. [5] and the Andean clade of Ericaceae that hosts cavendishioid ectendomycorrhizae [6], [7].

The presence of hyphal structures within hair roots of the Ericaceae attracted attention to the potential mycorrhizal partners already at the beginning of the twentieth century [8], [9] but it was not until 1973 that Pearson and Read isolated the first definite (and so far best-researched) ErM fungus, the ascomycete *Rhizoscyphus* (syn. *Hymenoscyphus*) *ericae* [10] and subsequently demonstrated bi-directional carbon/phosphorus transport between *R. ericae* and *Calluna vulgaris* [11]. Though at that time most investigations focused on potentially ericoid mycorrhizal ascomycetes readily isolated from Ericaceae roots, it soon became apparent that Ericaceae also host basidiomycetes [12], [13], [14], [15]. A physiologically active relationship between some basidiomycetes, most notably *Clavaria* spp., and Ericaceae was hypothesized [16] but only partly corroborated. For example, Englander and Hull [17] detected bidirectional carbon/phosphorus transport between Ericaceae and *Clavaria* sp. but nonetheless concluded that this could have been also due to a saprotrophic or necrotrophic, rather than mycorrhizal, relationship. Ericoid mycorrhiza thus largely remained as a domain of ascomycetes [1], [3] and observed associations of ericaceous roots with basidiomycetes were generally considered as “casual”, i.e., non-mycorrhizal [18].

This paradigm shifted after Berch *et al*. [19] and Allen *et al*. [20] reported that the majority of ericaceous hair roots under investigation contained DNA of basidiomycetous Sebacinales. Subsequent molecular-based studies confirmed that *Sebacina* spp. are the primary basidiomycete taxon detected in ErM roots [21], [22], [23], [24] and in 2006, Setaro *et al*. demonstrated that Sebacinaceae formed cavendishioid ectendomycorrhiza [7]. Additional basidiomycetes, such as relatives of white-rot fungi [24], [25] and various ectomycorrhizal (EcM) fungi [21], [26] have been detected sporadically with isolation- or molecular-based approaches, with unconfirmed symbiotic status. It remains to be shown whether these observations comprise true ericoid mycorrhizal or casual associations, because Ericaceae host a wide range of mycobionts including root endophytes with affinity to common soil saprobes [27].

Physiological aspects of ErM fungi and their interactions with host plants have been charactarized entirely from studies of commonly isolated ascomycetes, such as *R. ericae*, and *Oidiodendron maius* [28]. ErM ascomycetes are known to provide host plants with access to various otherwise inaccessible organic nutrients, including peptides [29], proteins [30], chitin [31] and plant, fungal, mycorrhizal and potentially protist necromass [32], [33], [34], protection against various abiotic stress agents such as heavy metal toxicity [35], and are hypothesized to link Ericaceae with neighboring EcM plants through shared mycorrhizal mycelium [36]. Conversely, host adaptations conferred by putative ErM basidiomycetes are currently unknown but are speculated to include a shared common mycelial network, providing for bi-directional carbon and nutrient transport between ErM and EcM plants [21]. In addition it is now known that numerous EcM basidiomycete genera possess Class II peroxidase-encoding genes, once considered exclusive to saprotrophic, white-rot basidiomycetes [37]. Therefore it is plausible that ErM basidiomycetes confer host adaptations that are distinct from or complementary to those provisioned by ErM ascomycetes. However, elucidating the nature of host interactions and potential function of putative ErM basidiomycetes requires detailed morphological observation in combination with study under controlled conditions, which to this point are lacking [4].

During the course of a study of a semi-natural *Vaccinium myrtillus* production in mid-Norway, we observed clamp-bearing hyphae forming dense sheaths around terminal parts of healthy ericaceous hair roots that colonized rhizodermal cells intracellularly in a manner typical for ericoid mycorrhizal symbiosis. Occurrence of this so far unreported colonization pattern (hereafter referred to as *sheathed ericoid mycorrhiza* and abbreviated as *sheathed ErM*) was similar to the colonization frequency of ascomycete ErM and dark septate endophytic fungi (unpublished results). Sheathed ErM were subsequently found in Ericaceae roots in a nearby Norway spruce (*Picea abies*) boreal forest from which live *V. myrtillus* shrubs used in the plantation were obtained. Here we report i) sheathed ErM colonization levels in the semi-natural *Vaccinium* plantation and in a natural mixed population of Ericaceae in the adjacent forest, ii) morphological, anatomical, and ultrastructural characteristics of sheathed ErM, iii) the identity of the mycobiont forming sheathed ErM as determined by phylogenetic analyses of three rDNA genes, iv) results of a series of *in vitro* experiments evaluating the ability of the mycobiont to form sheathed ErM and its impact on the growth of ericaceous and ectomycorrhizal host plants, and v) the lignocellulolytic potential of the basidiomycetous mycobiont relative to that of commonly isolated ErM ascomycetes.

## Materials and Methods

### Root Sampling

Root samples originated from a semi-natural European blueberry (*Vaccinium myrtillus* L.) plantation and a nearby forest. Permits were unnecessary because the plantation is maintained by the Bioforsk Grassland and Landscape Division (BGLD) and legal regulations do not restrict sampling of blueberry roots outside of natural reserves in the European Union. The plantation is located at BGLD, Kvithamar, Stjørdal in mid-Norway (N 63°29.417′, E 10°52.579′; 37 m a. s. l.) and was established in July 2008 by transplanting forest ground mats. The mats (plants and adhering soil, approx. 40×30×15 cm) of blueberry with some co-occurring cowberry (*Vaccinium vitis-idaea* L.) were taken from a regenerating Norway spruce (*Picea abies* (L.) Karst.) stand located at the foothill of Forbordfjellet (N 63°31.125′, E 010°53.287′; approx. 400 m a. s. l.). The forest soil is a typical podzol, with a layer of humus overlying mineral soil of sand and gravel. Mats were transplanted into cultivated soil (silt loam 0–26 cm, loam 26–30 cm and clay loam deeper than 30 cm) in the Kvithamar plantation. Before transplanting, a 10-cm layer of commercially available peat was incorporated into the soil to add organic matter, which also reduced the pH of the upper 10 cm of soil from 5.9 to 4.8.

A first set of root samples was randomly collected from six different microsites within the plantation in October 2010. A second set was collected from twelve microsites within the plantation in May 2011. A third set of root samples was taken in May 2011 as a random sample of naturally established Ericaceae growing adjacent to voids left after *Vaccinium* mats were excised to establish the plantation in 2008. Samples in 2011 were collected as entire plants, allowing roots to be traced to the plant species. In the forest, all co-occurring Ericaceae were sampled. Upon receipt in the laboratory, roots were washed free of the adhering substrate and stored at 5°C until processed. The initial set of samples from 2010 was divided into two parts, the first for assessment of fungal colonization and isolation of mycobionts, while the latter samples collected in 2011 were assessed for colonization only.

### Anatomy and Morphology of Sheathed Ericoid Mycorrhizae

Healthy-looking turgescent roots with developed clamped hyphal sheath were subjected to light microscopy and scanning electron microscopy (SEM). For light microscopy, roots were carefully cleaned from adhering soil and either directly placed in water on slides or retained for preparation of paraffin thin sections according to Pazourková [38]. An Olympus BX60 microscope equipped with DIC was used to screen the roots at 400X and 1000X magnification. Graphic documentation was modified for clarity in Paint.NET as needed. SEM photographs of sheathed roots were taken in the Olympus ESEM™ mode at low temperatures (−6°C to −3°C) using a FEI Quanta 200 microscope.

### Colonization Levels of Sheathed Ericoid Mycorrhiza *in situ*

*In situ* sheathed ErM colonization was quantified by mounting randomly selected hair roots (5–10 mm in length) on glass slides and counting the number of sheathed ErM roots; 1936, 1861 and 969 hair roots from the first (2010), second (2011 plantation) and third (2011 forest) set of root samples, respectively, were examined by light microscopy as described above. Clearing and staining procedures were not used because sheathed ErM roots were hyaline or only lightly pigmented and able to be distinguished from non-sheathed-ErM roots at 400–1000X magnification.

### Isolation of Mycobionts

30 sheathed roots, each approx. 5 mm in length, were selected from the first set of roots by examination at 200–400X under the compound microscope, surface-sterilized in 10% SAVO (household bleach, 4.5% available chlorine) for 30 seconds, rinsed three times in sterile water, and placed on modified Melin Norkrans agar (MMN) amended with 4 mg per L of benomyl (Sigma-Aldrich) to suppress the growth of most ascomycetes. Additional approx. 150 randomly selected (i.e., not screened *a priori* for sheathed ErM) hair roots were surface-sterilized and placed on benomyl-amended MMN. All roots were incubated in the dark at 20°C for 21 days. Emerging mycelia were grouped according to colony morphology and color, presence of clamped hyphae and growth rate and sub-cultured onto MMN without benomyl.

### Identification of the Isolated Mycobionts

DNA was extracted from the isolates representing distinct morphological groups using a SIGMA Extract-N-Amp™ Plant Kit (Sigma-Aldrich) following the manufacturer’s instructions. Nuclear ITS1-5.8S-ITS2 rDNA region was amplified using the ITS1F/ITS4 and ITS1/ITS4 primer pairs [39], [40]. This approach did not allow for identification of all obtained isolates, therefore other primer pairs for rDNA region were used: ITS1F/LB-W, NL4, LR6; ITS5/ITS4, LR6; NSA3/NLC2; NSI1/NLB3, NS24; ITS1/LR6, LB/W [41], [42]. PCR thermal cycling parameters were as follows: an initial denaturation step of 4 min at 94°C, 35 cycles consisting of a denaturation step at 94°C for 30 s, annealing at 55°C for 30 s, extension at 72°C for 70 s, and final extension at 72°C for 10 min. The length, quality, and quantity of the PCR products were checked by gel electrophoresis (1% agarose). PCR products were purified and sequenced by Macrogen Inc. (Seoul, Korea) using primers ITS1, ITS1F, ITS4, LB-W, LROR, NL1, NL4 and LR6 for ITS-LSU rDNA region and NS1, NS3, NS4, NS5 and NS24 for SSU rDNA [40], [43]. Arbitrary primed PCR by using the sequence of M13 minisatellite DNA with the primers M13-core (5′-GAGGGTGGCGGTTCT-3′), M13 (5′-TTATGTAAAACGACGGCCAGT-3′) and microsatellite primers 834c (5′-(AG)_8_ CG-3′) and 834t (5′-(AG)_8_ TG-3′) following methods of Nováková *et al.* ([44]; see Tab. S1) were used to confirm conspecificity of two isolates of the basidiomycetous mycobiont forming sheathed ErM (see below).

Similarity search was performed using BLASTn to find closest matches in GenBank. This procedure revealed sufficient taxonomic affinities (≥96% sequence identity) for all isolates except the basidiomycetes JPK 87 and JPK 90. The latter was subjected to phylogenetic analyses, based on the sequences of its SSU, 5.8S and LSU regions of nrDNA. The matrix produced by Matheny *et al*. [45] was used as a reference and the alignment and taxonomic sampling was modified manually in Bioedit 7.09 [46]. The matrix was completed through the addition of members of Gloeophyllales [47] and Amylocorticiales and Jaapiales [48] (Tab. S2). To filter both gaps and variable regions, we used Gblocks version 0.91b [49] with less stringent selection allowing smaller final blocks and gap positions within the final blocks. Maximum likelihood (ML) searches were conducted in PhyML 3.0. [50], via the Montpelier online server (http://www.atgc-montpellier.fr/phyml/) with 500 bootstrap replicates and in RAxML 7.2.7 [51], via the Cipres Portal (http://www.phylo.org/sub_sections/portal/) with 1000 bootstrap replicates. Bayesian searches (MB) were conducted with MrBayes 3.0 [52] and ten million replicates estimated together with burn-in value in Tracer v1.5 [53]. In the RAxML, individual α-shape parameters, GTR-rates, and base frequencies were automatically estimated for each of the three partitions. Parameters for PhyML and MB were estimated in jModeltest 0.1.1 [54],[55] which proposed a general time-reversible substitution model (GTR + G + I) as best fitting all the three partitions. Three data partitions were recognized, and the model parameters for each partition were estimated separately for MB analyses, whereas a single matrix was used for PhyML which does not allow partitioning of the data.

### Resynthesis Experiments

The methodology used for determining the mycorrhizal status of fungi obtained from Ericaceae roots varies among published reports, which makes comparisons of their results difficult [56]. Therefore, we evaluated four different *in vitro* resynthesis systems. All experiments had non-inoculated controls that were treated in the same manner as inoculated plants. The experiments were maintained in a growth chamber under a 21°C, 16-h light and 15°C, 8-h dark cycle and irradiation of 200 µmol m^–2^ s^–1^. Autoclaved peat used in some experiments was confirmed sterile by plating on nutrient agar.

In the first experiment, two-month-old axenic cowberry seedlings and 4 mm^2^ of mycelium cut from the margin of a fungal culture were placed together in 5-cm diam. Petri dishes containing MMN with 0.1% (w/v) glucose and 1% agar. Besides the basidiomycetes JPK 87 and JPK 90, the ErM ascomycete *Oidiodendron maius* Barron isolate OMA-1, the ascomycetes *Pochonia bulbillosa* (W. Gams & Malla) Zare & W. Gams (JPK 74) and a Pleosporales sp. (JPK 78), and two other basidiomycetes, *Mycena galopus* (Pers.) P. Kumm (JPK 75) and *Galerina* sp. (JPK 77), were included for comparison. Isolates other than *O. maius* originated from the root samples collected in October 2010 that were plated on MMN + benomyl agar (Tab. 1). Mycorrhizal resynthesis dishes were sealed with air-permeable plastic film and incubated in the growth chamber. After six weeks, entire root systems of the seedlings were harvested and examined for ErM colonization as the percentage of rhizodermal cells colonized.

**Table 1:** Identities of the isolates obtained in this study.

Isolate #	Identity	Source*(a)*	Primerpair	Length (bp) GenBank#	Closest matches *(b)*	Maximal Identity (%)
**JPK 74**	***Pochonia bulbillosa*** (Ascomycota)	RR	ITS1F/ITS4	344 JQ926165	AB378551	100
					EU999952	100
**JPK 75**	***Mycena galopus*** (Basidiomycota)	RR	ITS1F/ITS4	642 JQ926166	HM240534	99
					JF908484	99
**JPK 76**	***Nectriaceae*** ** sp.** (Ascomycota)	RR	ITS1F/ITS4	324 JQ926167	HM036602	100
					EF601613	100
**JPK 77**	***Galerina*** ** sp.** (Basidiomycota)	RR	ITS1F/ITS4	437 JQ926168	AJ585473	96
					AJ585471	96
**JPK 78**	**Pleosporales sp.** (Ascomycota)	RR	ITS1F/ITS4	493 JQ926169	JF740264	100
					HQ248194	89
**JPK 87 (CCF 4139)**	no similarity found (Basidiomycota)	SR	NL1/LR6	1081 HE802996	-	<80%
**JPK 89**	**Sebacinales sp.** (Basidiomycota)	SR	ITS1F/ITS4	611 JQ926170	AY112923	98
					FN663149	88
**JPK 90 (CCF 4138)**	no similarity found (Basidiomycota)	SR	NSI1/LR6	3526 HE573028	-	<90%

***(a)*** RR  =  isolation from random root samples, SR  =  isolation from sheathed ericoid mycorrhizae (see Materials and Methods);

***(b)*** preference was, where possible, for matches with a scientific name derived from a deposited culture or a fruit body with a deposited voucher. CCF  =  Culture Collection of Fungi, Faculty of Science, Charles University in Prague, Czech Republic.

The second experiment utilized the soil agar mycorrhizal resynthesis method described by Leake and Read [18]. Briefly, 12 ml of molten 0.8% agar amended with 0.1% activated charcoal (Sigma-Aldrich) was poured over twice-autoclaved, moist, peat-based potting soil (0.6 g dry weight) in 9-cm Petri dishes. After the media solidified, half of the agar was removed. Two blueberry and two cowberry seedlings were placed equidistantly along the upper margin of the soil agar that remained in each dish. A 4 mm^2^ plug of actively growing mycelium of *M. galopus* JPK 75, *Galerina* JPK 77, and the basidiomycetes JPK 87 and 90 was placed 2 mm below each seedling; uninoculated control dishes received a 4 mm^2^ plug of sterile MMN agar. There were 4 seedlings per plant species × fungal isolate combination. The dishes were sealed with two layers of air-permeable plastic film, covered with aluminum foil over the roots and held in the growth chamber for 83 days, after which seedlings were harvested and total shoot and root length and dry shoot weight were recorded. ErM colonization of 50 rhizodermal cells of twenty randomly selected, 5- to 10-mm-long hair roots ( = 1000 rhizodermal cells) per plant was assessed at 400 to 1000X magnification using the Olympus BX60 microscope.

The third experiment employed autoclaved soil overlying MMN modified with 0.8% agar (w/v) and 0.05% glucose (w/v) and malt extract omitted. A 15-ml volume of medium was added to 18-cm length × 2 cm-diameter test tubes and inoculated with a 1 mm^2^ mycelial plug (same fungal isolates as in the second experiment) after the media solidified. After 10 days, twice-autoclaved, moist, peat-based potting soil was added to the test tubes to a depth of 1 cm, and one blueberry or cowberry seedling was transferred to each tube (modified from [57]). Each plant species × fungal isolate combination and non-inoculated control was replicated in three test tubes. The tubes were covered with two layers of air-permeable plastic film and aluminum foil at the bottom. After 90 days total shoot and root length and dry shoot weight were measured. ErM colonization was assessed on a cell-by-cell basis, as per the second experiment. Roots that developed an ErM sheath were surface sterilized as above and plated on MMN to satisfy Koch’s postulates. Additionally, several sheathed ErM roots were retained for examination by transmission electron microscopy (TEM). Root segments were fixed in 5% glutaraldehyde solution in 0.1 M phosphate buffer (pH 7.2) and post-fixed in 2% OsO_4_ in 0.1 M phosphate buffer. The samples were then dehydrated in an increasing concentration of ethanol, including a contrasting step with 1% uranyl acetate. Infiltration was performed in increasing concentration series of propyleneoxide and Spurr Resin. Samples were embedded into Spurr resin and ultrathin sections (70 nm) were cut and contrasted with uranyl acetate and lead citrate. Photographs were acquired with a digital TEM camera (Veleta, Olympus) using a JEOL 1011 microscope.

The fourth experiment employed Norway spruce seedlings to test the interaction of the basidiomycetes JPK 87 and JPK 90 with a model ectomycorrhizal host plant. It utilized the same media as the third experiment but without peat soil. It was poured in 12×12 cm Petri dishes and half was removed after solidification. The other half was inoculated with 5-mm-diam. mycelial plugs (the basidiomycetes JPK 87 and JPK 90, the *Rhizoscyphus ericae* (Read) Korf & Kernan isolate RER-2 and the *Hebeloma bryogenes* Vesterh. isolate HBR-1) and incubated in the dishes for three weeks at room temperature in the dark. The *R. ericae* RER-2 isolate was chosen as a representative of a typical ascomycetous ErM fungus, the *H. bryogenes* HBR-1 isolate as a representative of basidiomycetous EcM fungi, as it readily forms EcM symbiosis with spruce under growth chamber conditions [58]. Two blueberry + one spruce seedling were introduced into each dish, which were then sealed with two layers of the plastic film and their lower parts were covered with aluminum foil. There were six dishes for the basidiomycetes JPK 87 and JPK 90 and one for *R. ericae* RER-2, *H. bryogenes* HBR-1 and a non-inoculated control. The plants were harvested after 10 weeks and their roots were screened for fungal colonization. Semi-thin hand sections were made from spruce root tips using a razor blade to confirm presence of the Hartig net.

Fungal isolate and plant species effects on plant growth in resynthesis experiments were assessed by two-way ANOVA, using a log- or cubic-root-transformation to satisfy ANOVA assumptions and Tukey HSD for mean separation. Pearson product-moment correlation coefficient (r) was used to evaluate the relationship between ErM colonization and plant growth parameters. All analyses used a type I error rate of α = 0.05 and were conducted in SAS 9.3 (SAS Institute Inc., USA).

### Lignocellulolytic Test

MMN medium, containing either 0% or 0.1% (w/v) glucose, and 0.8% (w/v) agar was added at a volume of 4.5 ml to glass vials. An additional 0.5 ml of MMN agar with cellulose azure (1.5% w/v; Sigma-Aldrich C1052; cellulose bound to Remazol Brilliant Violet 5R dye) was then pipetted onto solidified agar at the same glucose concentration as the underlying medium, thereby providing cellulose or cellulose plus glucose as a C source for inoculated fungi. One plug of actively growing mycelium (4 mm^2^) of *R. ericae* RER-2, *O. maius* OMA-1, or the basidiomycetes JPK 87 or JPK 90 was placed in the center of the agar; control vials were not inoculated. At each glucose concentration, two replicate vials were included for each fungal strain and two non-inoculated vials served as controls. Vials were sealed and incubated at 22°C for two months. This test provides a simple, effective assay for both cellulolytic and ligninolytic abilities of the screened fungi; release of azure dye indicates cellulose degradation while clearing of azure dye, whether or not it is released from cellulose azure, indicates activity of ligninolytic enzymes [59].

## Results

### Anatomy and Morphology of Sheathed Ericoid Mycorrhiza

**Figure 1:**
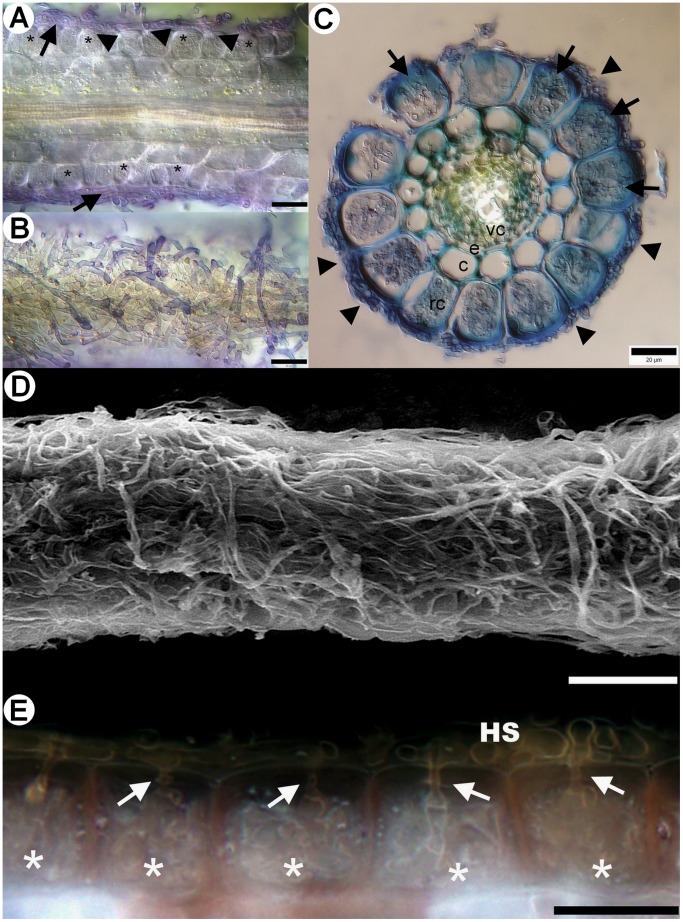
Morphological and anatomical characteristics of sheathed ericoid mycorrhiza from field-collected European blueberry (*Vaccinium myrtillus*) roots. 1A) Longitudinal section through sheathed ericoid mycorrhiza. The hyphae forming the sheath (arrows) penetrate rhizodermal cell walls (arrowheads) and form dense coils typical for ericoid mycorrhiza (asterisks). DIC, stained with trypan blue, bar  = 20 µm. 1B) Surface view of the same sheathed ericoid mycorrhiza displaying the structure of a dense hyphal sheath covering the hair root. DIC, stained with trypan blue, bar  = 20 µm. 1C) Cross section of sheathed ericoid mycorrhiza. The hair root is covered by a hyphal sheath (arrowheads), its rhizodermal cells (rc) are filled with dense hyphal coils (arrows). The mycobiont never advances to the cortex/exodermis (c), the endodermis (e) or the vascular cylinder (vc). DIC, stained with trypan blue, bar  = 20 µm. 1D) Surface view of sheathed ericoid mycorrhiza showing a dense hyphal sheath. SEM, bar  = 50 µm. 1E) Detail of a longitudinal section of sheathed ericoid mycorrhiza. Hyphae forming the hyphal sheath (HS) penetrate rhizodermal cells (arrows) and form coils typical of ericoid mycorrhizal symbiosis (asterisks). DIC, bar  = 20 µm.

**Figure 2:**
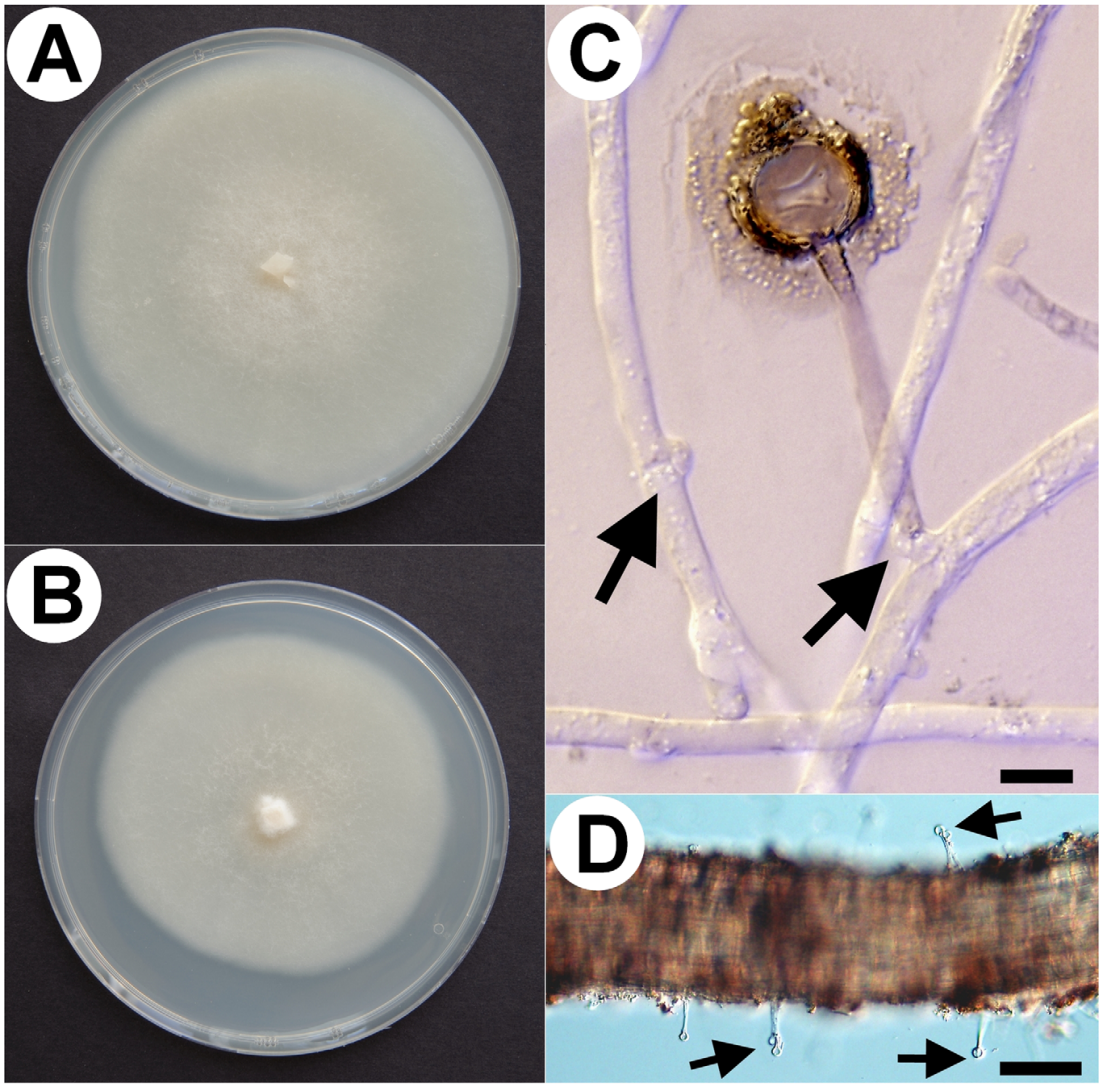
Some morphological characteristics of the novel basidiomycete (isolates JPK 87 and 90). 2A) JPK 87 culture grown in a 9 cm diam. Petri dish on MMN for three weeks. 2B) JPK 90 grown under the same conditions. 2C) Capitate cystidium excreting a brownish substance around its apical part. Note abundant clamp connections (arrows) produced *in vitro* on the mycelium of JPK 87. DIC, bar  = 10 µm. 2D) Similar capitate cystidia were formed of sheathed ericoid mycorrhizal roots under natural conditions (arrows). *Vaccinium* sp. root, DIC, bar  = 50 µm.

**Figure 3:**
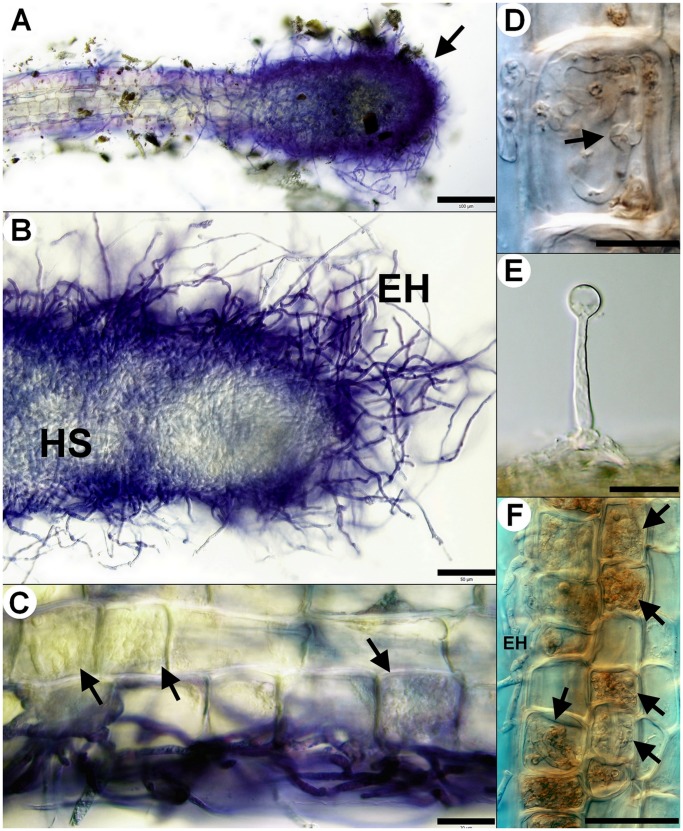
Morphological and anatomical characteristics of sheathed ericoid mycorrhiza synthesized *in vitro* and observed under natural conditions. Sheathed ericoid mycorrhiza was synthesized *in vitro* between the basidiomycetous mycobiont (strains JPK 87 and JPK 90) and European blueberry (*Vaccinium myrtillus*) seedlings or observed in European blueberry hair roots collected in field. 3A) Terminal part of hair root is covered by a dense hyphal sheath (arrow). JPK 87, DIC, stained with trypan blue, bar  = 100 µm. 3B) Detail of a dense hyphal sheath (HS) covering terminal part of a hair root. Note the extensive extraradical hyphae (EH). JPK 90, DIC, stained with trypan blue, bar  = 50 µm. 3C) Dense intracellular hyphal coils (arrows) developing in the rhizodermal cells below a hyphal sheath. JPK 90, DIC, stained with trypan blue, bar  = 20 µm. 3D) Clamp connection (arrow) formed by the basidiomycete inside a rhizodermal cell. Roots from field, DIC, bar  = 10 µm. 3E) Terminally swollen capitate cystidium emerging from a sheathed ericoid mycorrhiza. Roots from field, DIC, bar  = 10 µm. 3F) Vigorous intracellular colonization (arrows) by the basidiomycete accompanied by numerous extraradical hyphae (EH). JPK 90, DIC, bar  = 50 µm.

A yet undescribed type of ericoid mycorrhizal association in *Vaccinium* spp. was observed in field-collected roots, that we designate here as sheathed ericoid mycorrhiza. Its most prominent characteristic is a dense layer of clamp-bearing hyphae over the surface of terminal parts of young hair roots (Fig. 1a–e). Hyphae comprising the sheath were of variable diameter and penetrated epidermal cells, forming dense hyphal coils typical for ericoid mycorrhizae (Fig. 1a, c, e). The intraradical hyphae were limited to rhizodermal cells and never advanced into the endodermis or vascular cylinder (Fig. 1a, c). Hyphal protrusions bearing terminally swollen parts emerged from the sheath in some roots (Figs 2d, 3e). These structures resembled capitate cystidia formed by some EcM basidiomycetes on the surface of colonized roots [60] and were morphologically identical to those observed in resynthesis trials with the basidiomycetes JPK 87 and JPK 90. Sheathed ErM colonization was limited to the lowest-order hair roots with an invariably pale tan-yellow pigmentation. By comparison, roots with non-clamped ErM hyphae or dark septate endophyte hyphae were most often pigmented. Morphological features of sheathed ErM did not differ between the plantation and the forest.

### Colonization Levels of Sheathed Ericoid Mycorrhiza *in situ*

In the first set of root samples, sheathed ErM colonization was 4±2% (mean ± SE, n = 6 soil cores, 1936 hair roots total, mix of *V. myrtillus* and *V. vitis-idaea*). The second sampling of twelve microsites in May 2011 revealed that sheathed ErM colonization was 6±5% of *V. myrtillus* and 4±2% of *V. vitis-idaea* roots (n = 12 soil cores, 916 and 539 total *V. myrtillus* and *V. vitis-idaea* hair roots evaluated, respectively). In the nearby forest, sheathed ErM colonization was 2±1% of *V. vitis-idaea* (n = 4 microsites, 221 total hair roots evaluated), 0.5±0.6% of *V. myrtillus* (n = 4 microsites, 203 total hair roots evaluated), and 0% of *C. vulgaris* and *E. nigrum* (n = 4 microsites, 212 hair roots, and n = 3 microsites, 168 hair roots evaluated respectively).

### Isolation and Identification of the Mycobionts

Three isolates with distinct morphology and/or growth rate were obtained from 30 sheathed ErM roots plated onto benomyl-amended MMN (Tab. 1, source “SR”). One had hyphae without clamp connections (Sebacinaceae sp. JPK 89) while the other two had clamped hyphae (the basidiomycetes JPK 87 and JPK 90) and their morphology corresponded to the hyphae forming sheathed ErM, including capitate cytidia with a brownish, iridescent substance around the terminal portion (Fig. 2c), resembling those described by Larsson [61]. Sebacinaceae sp. JPK 89 lost its viability in culture shortly after the transfer from the original colony to MMN (in our experience Sebacinaceae are notoriously difficult to maintain in pure cultures); however, it is apparent that this clampless isolate did not form the respective symbiosis. In contrast, both clamped basidiomycetes JPK 87 and JPK 90 grew well on MMN (the former slightly faster) producing dense whitish colonies (Fig. 2a, b). These strains were deposited in Culture Collection of Fungi (Faculty of Science, Charles University in Prague, Czech Republic) under accession numbers CCF 4138 ( =  JPK 90) and CCF 4139 ( =  JPK 87), respectively and are available upon request from the collection curator (kubatova@natur.cuni.cz) or the corresponding author. Another five isolates obtained on the benomyl-amended MMN from the approx. 150 randomly selected hair roots were sequenced and identified according to BLASTn search. Two were basidiomycetes with clamped hyphae (JPK 75 =  *Mycena galopus* and JPK 77 =  *Galerina* sp.), while the other three were ascomycetes with simple septate hyphae (JPK 74 =  *Pochonia bulbillosa*, JPK 76 =  *Nectriaceae* sp. and JPK 78 =  Pleosporales sp.) (Tab. 1, source “RR”).

In contrast to *Galerina* sp. JPK 77 used as a reference, the basidiomycetes JPK 87 and JPK 90 repeatedly failed to amplify with the most common ITS1F/ITS4 and ITS1/ITS4 primer pairs and also failed with ITS1F/LB-W, NL4, LR6; ITS5/ITS4, LR6; NSA3/NLC2; and NSI1/NLB3. The taxonomic resolution of the basidiomycetes JPK 87 and JPK 90 was still not sufficient when their LSU rDNA was amplified with the LROR/LR5 primer pair. Eventually, we succeeded to amplify the entire ITS-LSU rDNA region (ITS1-5.8S-ITS2 rDNA and D1-D4 domain of LSU) and SSU rDNA of the basidiomycete JPK 90 using primer pairs ITS1/LR6, ITS1/LB-W and NS1/NS24, respectively. ITS regions of the basidiomycete JPK 90 showed only low similarity to deposited sequences, while separate searches of the three ribosomal genes showed weak similarity to diverse spectrum of species from various Agaricomycotina orders (5.8S, 168 bp, maximal similarity 90%; LSU, 1089 bp, 86%; SSU, 1749 bp, 86%). Although differing in growth rate and production of capitate cystidia (data not shown), the basidiomycetes JPK 87 and JPK 90 were shown to be conspecific by the minisatellite analysis (Tab. S1). Because its ITS sequence was available, the latter was chosen as a representative of the novel basidiomycete and its sequence (18S, ITS1, 5.8S, ITS2 and 28S rDNA) was submitted to GenBank under accession number HE573028. The 28S rDNA sequence of the basidiomycete JPK 87 was submitted under acc. no. HE802996.

**Figure 4:**
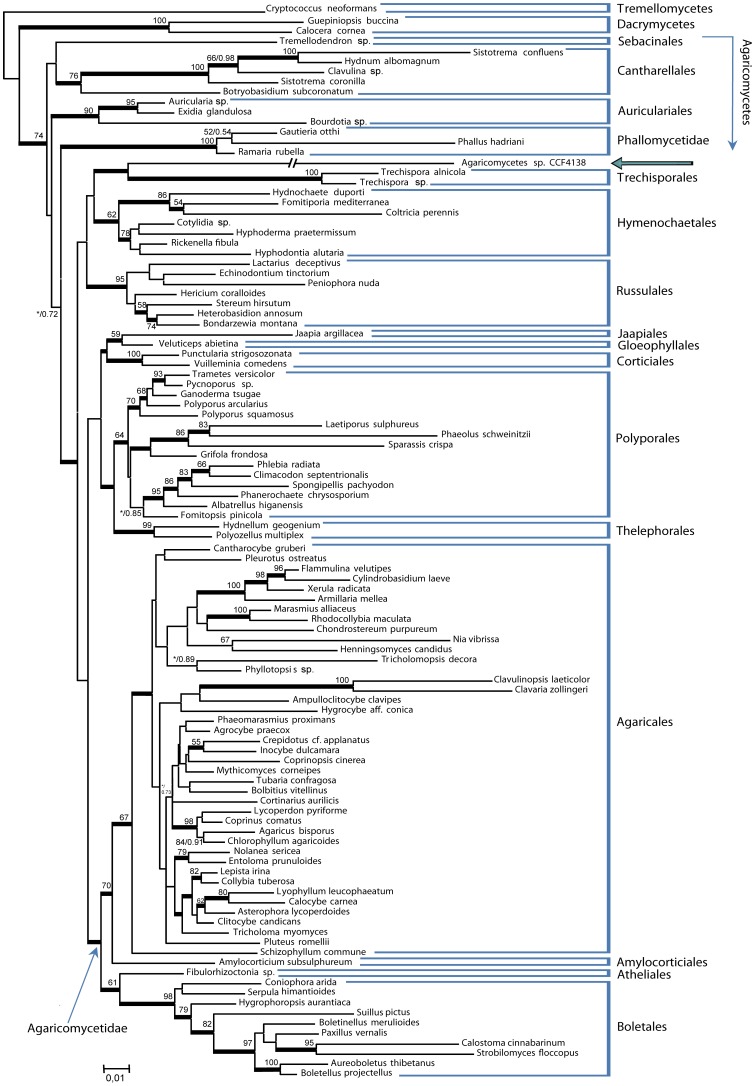
Phylogenetic relationship of 106 members of Agaricomycetes, including a representative of the novel basidiomycete. The representative of the novel basidiomycete (JPK 90 =  Agaricomycetes sp. CCF 4138) is marked with an arrow, the relationship is inferred from nuclear LSU, SSU and 5.8S rDNA genes; the new lineage forms a sister clade to Trechisporales and Hymenochaetales. Topology, branch lengths and bootstrap values above branches are from PhyML analyses. Thickened branches indicate Bayesian posterior probability ≥0.95. The branch leading to Agaricomycetes sp. CCF 4138 is approx. three-times shortened.

The final cured dataset for phylogenetic analyses of the basidiomycete JPK 90 contained 106 taxa and 2727 positions (76% of the original 3572 positions). Both ML and MB analyses generated topologies in agreement with published rDNA based phylogenies of the Basidiomycota [61], [62], [63]. ML versus MB differed in the position of the Auriculariales, which were placed as most related to the Dacrymycetes in MB analyses or more derived in ML phylogenies. The basidiomycete JPK 90 (CCF 4138) formed a long branch residing as sister to Trechisporales (PP = 0.98) in MB analyses (Fig. 4), but being inconsistently clustered with Trechisporales, Hymenochaetales or Russulales in ML trees.

### Resynthesis Experiments

**Figure 5:**
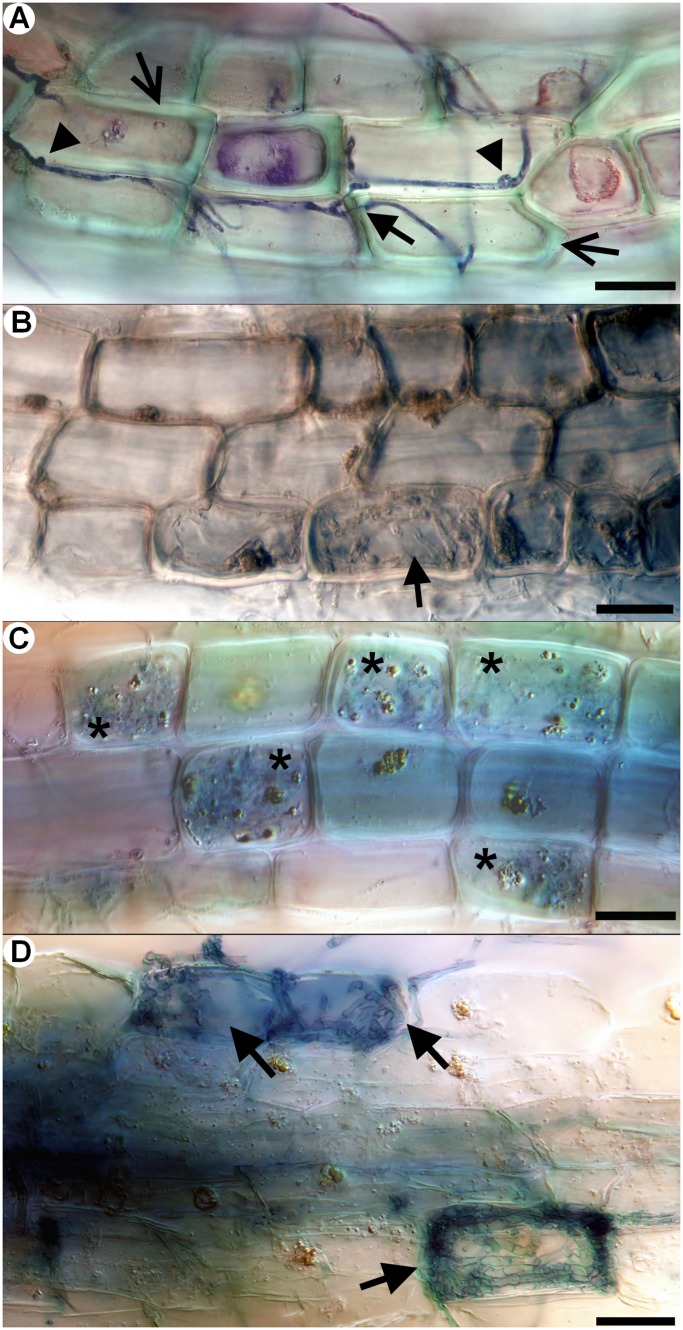
*In vitro* resynthesis between European blueberry (*Vaccinium myrtillus*) and saprotrophic, ericoid mycorrhizal and ectomycorrhizal fungi. Saprotrophic *Galerina* sp. JPK 77, ericoid mycorrhizal *Rhizoscyphus ericae* RER-2 and ectomycorrhizal *Hebeloma bryogenes* HBR-1 were used in the resynthesis test. The hair roots were stained with trypan blue (except 6b) and observed with DIC. All bars correspond to 20 µm. 4A) *Galerina* narrow hyphae pass through blueberry rhizodermal cells. Note clamp connections (arrowheads) and thin hyphae (arrow) penetrating thickened host cell walls (open arrows). 4B) Loose hyaline loops (arrow) rarely formed by *Galerina* in blueberry rhizodermal cells. 4C) Ericoid mycorrhizae (asterisks) formed by the typical ericoid mycorrhizal fungus *R. ericae*. 4D) Intracellular colonization formed by the ectomycorrhizal fungus *H. bryogenes* (arrows).

The seedlings on plain MMN with 10X diluted sugars prospered regardless of fungal inoculation. Seedlings inoculated with *P. bulbillosa* JPK 74 and Pleosporales sp. JPK 78 had healthy turgescent roots with no intracellular hyphal colonization. *O. maius* OMA-1 formed extensive ErM colonization of the lowest order hair roots, which reached up to 100% of the rhizodermal cells. *M. galopus* JPK 75 produced no intracellular colonization and roots of the inoculated seedlings remained turgescent and healthy, while those inoculated with *Galerina* sp. JPK 77 sometimes showed signs of degradation; root initials surrounded by clamped mycelium were darkened with cells partially or totally collapsed. Some roots inoculated with *Galerina* sp. JPK 77 had rhizodermal cells intracellularly colonized by clamped coiled or non-coiled hyphae. The latter often passed from one cell to other forming thin penetrating hyphae when crossing the host cell wall (Fig. 5a). The basidiomycete JPK 90 intracellularly colonized turgescent rhizodermal cells in a manner typical for ErM symbiosis and often formed loose hyphal sheaths/numerous running hyphae on the surface of the colonized roots (Fig. 3f). Clamp connections were sometimes visible within individual rhizodermal cells (Fig. 3d), a situation sometimes observed in Ericaceae roots in nature [64]. Intriguingly, the basidiomycete JPK 87 formed hyphal sheaths around the inoculated roots, but produced no intracellular colonization.

**Figure 6:**
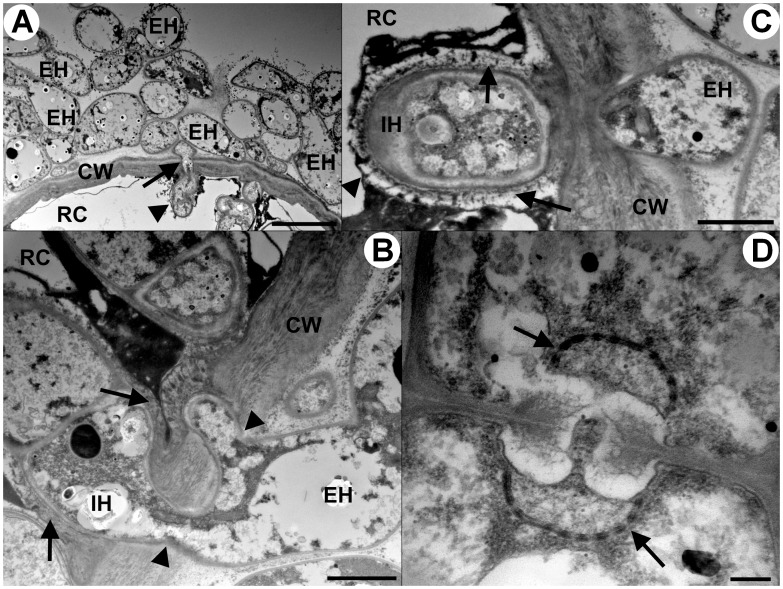
Transmission electron microscopy of sheathed ericoid mycorrhizae synthesized *in vitro* between the basidiomycete JPK 87 and *Vaccinium vitis-idaea.* 1A) Transversal section through a hyphal sheath, showing abundant extraradical hyphae (EH); a hypha (arrow) narrows its diameter while penetrating the host cell wall (CW) and then expands again in the lumen of a host rhizodermal cell (RC). The intracellular hypha is surrounded by the host plasma membrane (arrowhead). Bar  = 5 um. 1B) Thick extraradical hypha (EH) penetrates the cell wall (CW) of a host rhizodermal cell (RC) at two entry points (arrowheads). The intracellular hypha (IC) is surrounded by a material apparently derived from the host cell wall (arrows) deposited in the space below the host plasma membrane. Bar  = 1 µm. 1C) Extraradical hypha (EH) penetrates the host cell wall (CW), its intracellular phase (IH) is surrounded by the host plasma membrane (arrowhead) and the cell wall-derived material (arrows). Bar  = 1 µm. 1D) A dolipore with perforated parenthesomes (arrows) of an intracellular hypha. Bar  = 0.2 µm.

On soil agar, sheathed ErM formed by the basidiomycetes JPK 87 and JPK 90 occurred in, but were not limited to, root apices and did not appear to follow a predictable pattern among root orders. The extent of intracellular colonization among inoculated plants was highly variable (coefficient of variation  = 1.7). Significant correlations between ErM intracellular colonization and shoot length (r = 0.74, *p* = 0.002) and shoot weight (r = 0.54, *p* = 0.04), along with negligible shoot growth of non-(ErM)-colonized and non-inoculated control plants, indicate that colonization by the basidiomycetes JPK 87 and JPK 90 positively contributed to shoot growth. *Galerina* JPK 77 formed loose hyphal coils in less than 0.1% rhizodermal cells (Fig. 6b), while roots inoculated with *M. galopus* JPK 75 had no intracellular colonization.

Sheathed ErM colonization of *Vaccinium* roots by the basidiomycetes JPK 87 and JPK 90 also occurred on MMN overlain with soil. In the MMN section, the colonization process usually started at the apical part of the newest hair roots by embedding with loose hyphae, which eventually led to formation of very dense multilayered sheaths (Fig. 3a, b) accompanied with intracellular colonization of the rhizodermal cells (Fig. 3c). In the soil section, intracellular colonization was initiated >10 cells proximal to the root apex and ErM sheaths developed distally to these cells. Seedlings inoculated with the basidiomycetes JPK 87 and JPK 90 grew well with no signs of nutrient deficiency; in contrast, seedlings inoculated with *M. galopus* JPK 75 and *Galerina* sp. JPK 77 did not develop new shoots after transferring to the test tubes; leaves developed a yellowish-brown coloration and the seedlings eventually died. Koch’s postulates were fulfilled by recovery of the basidiomycete mycelium from sheathed ErM roots that was morphologically identical to that inoculated earlier. TEM confirmed that the hyphae forming the sheath also penetrate host cells to form intracellular coils (Fig. 6a). Penetrating hyphae had reduced diameter and subsequently became embedded in the host plasma membrane after passing through host cell wall (Fig. 6b, c). A plant-cell-wall-derived material was deposited in the space between the host membrane and the mycobiont cell wall (Fig. 6b, c). The basidiomycete possessed dolipores with perforate parenthesomes (Fig. 6d). Variability of intracellular ErM colonization was less (c. v. = 1.1) than in the soil agar system, and all but one of the inoculated plants became colonized. Shoot length, shoot weight, and root length of *Vaccinium* inoculated with the basidiomycetes JPK 87 and JPK 90 increased significantly relative to uninoculated (control) plants (*p*<0.001; Tab. 2), but did not differ significantly between the basidiomycetes JPK 87 and JPK 90 or *Vaccinium* species (*p*>0.05). There was no significant correlation between the level of intracellular ErM colonization and shoot length, shoot weight, or root growth (*p*>0.1), contrary to results obtained with the soil agar re-synthesis system.

**Table 2:** Effects of the basidiomycete on growth of *Vaccinium* sp.

	Total shoot length (mm)	Dry shoot weight (mg)	Total root length (mm)	Ericoid mycorrhizal colonization (% cells)
**JPK 87**	39.3 (3.6) **a** †	4.9 (0.2) **a**	79.3 (8.1) **a**	12.6 (4.7)
**JPK 90**	39.3 (5.8) **a**	4.8 (0.4) **a**	103.7 (23.2) **a**	22.1 (10.4)
**Not inoculated**	12.8 (1.2) **b**	1.2 (0.1) **b**	13.2 (4.7) **b**	0
ANOVA	*P*<0.001	*P*<0.001	*P*<0.001	‡

† Different letters indicate significant differences (*p*<0.001, Tukey HSD).

‡ Not analyzed.

The effect of the isolates JPK 87 and JPK 90 on shoot length, dry shoot weight, total root length, and ericoid mycorrhizal colonization of *Vaccinium myrtillus* and *V. vitis-idaea* seedlings grown in a modified Melin Norkrans agar medium overlain with sterile peat soil. Prior to ANOVA data were log- (total shoot length and dry weight) or cubic-root- (total root length) transformed; untransformed means (n = 6) are shown with standard error of the mean in parentheses. *V. myrtillus* and *V. vitis-idaea* did not differ (*p*>0.05) for all growth parameters and were pooled for analysis. ANOVA numerator and denominator degrees of freedom were 2 and 15, respectively.

In the resynthesis experiment with both ericaceous and EcM hosts, intracellular colonization levels by the basidiomycetes JPK 87 and JPK 90 was similar to that of *R. ericae* RER-2, which formed typical intracellular ericoid mycorrhizae (Fig. 5c), suggesting that under given experimental conditions, the ability of the basidiomycete to form ericoid mycorrhiza matched that of the prominent ErM fungus. The basidiomycetes JPK 87 and JPK 90 seemed to have no effect on the spruce seedlings; they looked healthy and grew well, without any signs of fungal colonization in the internal parts of their roots, which had morphology similar to the control non-inoculated plants, i.e., simple branching and abundant root hairs. On the other hand, spruce seedlings formed typical EcM symbiosis with *H. bryogenes* HBR-1, with a hyphal mantle around root tips and an intercellular cortical Hartig net. In such ectomycorrhizae, root hairs were absent. *H. bryogenes* HBR-1 also intracellularly colonized some turgescent blueberry rhizodermal cells, forming loose hyphal loops and microsclerotia-like structures (Fig. 5d), but such colonization level was very rare (<0.1% of rhizodermal cells) and did not resemble the typical ErM colonization pattern formed in this study by *O. maius*, *R. ericae* or the basidiomycetes JPK 87 and JPK 90.

### Lignocellulolytic Test

**Figure 7:**
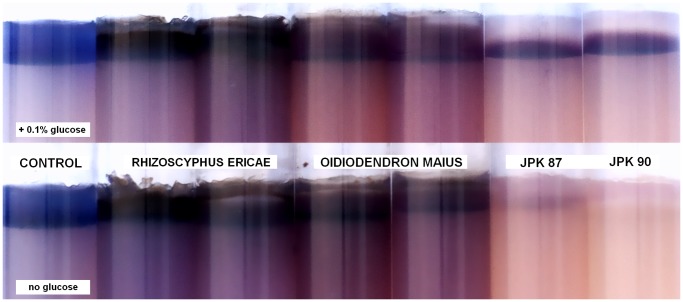
Lignocellulolytic test comparing enzymatic capabilities of ascomycetous ericoid mycorrhizal fungi with the novel basidiomycete. The ability to degrade cellulose azure of representatives of typical ericoid mycorrhizal ascomycetes (*Oidiodendron maius* and *Rhizoscyphus ericae*) was compared with the basidiomycetous isolates JPK 87 and JPK 90. This test provides a simple, effective assay for both cellulolytic and ligninolytic abilities of the screened fungi; release of azure dye from the upper part of the agar medium indicates cellulose degradation (*O. maius*, *R. ericae*) while clearing of azure dye indicates activity of ligninolytic enzymes (the basidiomycetes). In the upper row, the agar medium contains 0.1% (w/v) glucose, while glucose is absent in the lower row; it is evident, that the degree of clearing by the basidiomycetes depended on the initial glucose concentration. As complete clearing occurred only on media lacking glucose, we suggests that readily available carbon (e.g., from a host plant) may partly repress ligninolytic activity of the basidiomycetous isolates.

Dye in the overlying cellulose azure medium remained in place in the control non-inoculated vials. The azure dye diffused to the underlying agar in the presence of both ErM ascomycetes, indicating cellulose degradation, while it was partially or fully decolorized by the basidiomycetes JPK 87 and JPK 90. The degree of clearing by the basidiomycetes depended on the initial glucose concentration in the medium. Complete clearing only occurred on media lacking glucose, suggesting that readily available C (glucose) may partly repress ligninolytic activity of these isolates (Fig. 7).

## Discussion

### Saprotrophic and Ectomycorrhizal Basidiomycetes in Ericaceae Roots

A few non-sebacinoid basidiomycetes have been recently isolated from Ericaceae roots, with affinities to *Trechispora* (Trechisporales) [20] and *Irpex* [24] and *Trametes hirsuta* [25] (Polyporales). The symbiotic status of the *Trechispora* sp. strain was not investigated and the authors stated that it was “probably saprobic” [20]. The *Irpex* strain formed “loose, thin, hyaline coils” in *Vaccinium uliginosum* roots *in vitro* but this colonization pattern apparently differed from the typical ErM colonization produced by the other ascomycetous ErM isolates tested [24]. The *Trametes* strain formed “coil-like structures in the epidermal cells” of *Rhododendron fortunei* seedlings *in vitro* and the authors claimed that it showed positive effects on the seedlings. Unfortunately, these effects were not substantiated by any data presented and the colonization pattern was not documented except the brief description. Additionally, the recovery of the *Trametes* strain from *Rhododendron* roots was low (1 isolate out of 220) and its colonization potential in the *in vitro* test was “quite low”. *T. hirsuta* is a specialized wood decaying saprobe [65] which has not been previously reported from Ericaceae roots. In the current study, the most likely saprotrophic *Galerina* sp. isolate occasionally formed loose hyphal coils in *Vaccinium* rhizodermal cells *in vitro*. However, this pattern occurred at very low frequency and differed from that of typical ErM fungi, instead resembling loose intracellular hyphal coils formed by the soil saprobe *Geomyces pannorum* [66]. On the one hand, the ability of *Galerina* sp. to occasionally colonize *Vaccinium* roots indicates that under suitable circumstances, some saprotrophic basidiomycetes may coexist with Ericaceae as endophytes or weak pathogens. On the other hand, given the relatively short life span of delicate hair roots which become a suitable substrate for decomposers, it is evident that precautions must be taken before classifying associations between typical saprotrophic basidiomycetes and Ericaceae as mycorrhizal. These facts raise questions of whether “putative mycorrhizal fungi”, such as the *Trametes* strain isolated by Zhang *et al.* [25], are truly ericoid mycorrhizal.

Apart from saprobes, DNA of some ectomycorrhizal basidiomycetes has been recently detected in ErM roots [21], [26], [24]. However, culture-independent approaches detect also asymbiotic fungi, as root surface sterilization before DNA extraction does not necessarily destroy all traces of superficial or endophytic associates that may still be detected by PCR [67]. Some EcM basidiomycetes may potentialy colonize ericaceous roots internally, as here demonstrated for *Hebeloma bryogenes*, and this might explain occassional DNA detection in Ericaceae roots. However, the ecological significance of such colonization is questionable. In many ecosystems, Ericaceae typically form undergrowth of EcM trees and considerable efforts have been invested in determining whether ericoid mycorrhizal and ectomycorrhizal plants share mycorrhizal partners. While a functional mycelial link between ErM and EcM plant has to this point not been demonstrated, rich ecological evidence has accumulated to suggest that such liaisons appear improbable [68], [69], [70]. To conclude, there is currently no evidence to suggest that EcM basidiomycetes detected in Ericaceae roots form functional ericoid mycorrhizae. Congruently, both strains of the novel basidiomycete formed sheathed ericoid mycorrhiza with *Vaccinium*, yet did not form EcM symbiosis with Norway spruce.

### Sheathed Ericoid Mycorrhiza is a Novel Mycorrhizal Symbiosis in Ericaceae

In contrast to previous reports, this study demonstrates the first coherent evidence that a non-sebacinoid basidiomycete forms ericoid endomycorrhiza, meaning characteristic root-fungus symbiosis with the typical morphological properties, i.e., dense intracellular hyphal coils in healthy roots formed both *in vitro* and *in situ*. The symbiosis benefitted the host plants as evidenced by significantly enhanced growth in two independant trials. Moreover, sheathed ericoid mycorrhiza was observed under natural conditions at ecologically significant levels, a trait which has never been documented for any non-sebacinoid basidiomycete of Ericaceae. Our observation that sheathed ErM were limited to non-suberized and non-pigmented young roots implies that the association occurs in the portions of root systems that are most physiologically active, because heavy pigmentation of roots is often indicative of declines in metabolism, senescence of mycorrhizal structures, and onset of root dormancy or mortality [71].

The layered sheath formed by clamped hyphae and accompanied by extensive intracellular colonization of the rhizodermis, but without intercellular phases, appears to be a unique feature in Ericaceae. It is generally observed that ascomycetous ErM fungi do not produce developed extraradical hyphal mantles around colonized roots; so far the only described symbiosis of Ericaceae characteristically possessing hyphal mantles is cavendishioid ectendomycorrhiza. However, this symbiosis is formed by non-clamped hyphae of Sebacinales and its mantles are accompanied by intercellular fungal tissue resembling a Hartig net [62].

Interestingly, Massicotte *et al*. [5] presented a picture of a cross-section of a *Gaultheria procumbens* hair root embedded in a loose fungal mantle which may resemble sheathed ericoid mycorrhiza. However, the authors did not determine the identity of the respective mycobiont or remark about the possibility that this colonization pattern could have been produced by a basidiomycete. Sampling from a broader array of sites is needed to determine whether sheathed ericoid mycorrhizae occur outside of the area that we investigated in the current study.

### Potential Ecophysiological Functioning of Sheathed Ericoid Mycorrhiza

Within individual root systems, sheathed ErM had patchy distribution, being locally abundant but nearly absent outside the patches. The reason for such discontinuous distribution remains unknown, but might follow patchiness of a hypothetical substrate preferentially inhabited and decomposed by the basidiomycete. Ericaceae often dominate the boreal forest understory and heathlands where lignocellulosic residues accrue [72]. Relative to ligninolytic basidiomycetes, ErM ascomycetes have more limited capacity for lignin degradation [73], [74] due to attack of lignocellulose polymers via release of cellulases and hydroxyl radicals rather than true ligninases [75]. Two conspecific sheathed-ErM-forming basidiomycetes decomposed a model lignin compound that remained unaltered by ErM ascomycetes. This observation demonstrates that the ligninolytic potential of the novel ErM basidiomycete is similar to that of many specialist basidiomycete decomposers [76] including white-rot fungi generally regarded as the principle agents of lignin decomposition in forest ecosystems.

The sheathed ErM basidiomycete’s capacity for degrading complex aromatic polymers may allow for proliferation in highly lignified debris, while ErM association provides a direct conduit for transfer of nutrients therein to the host plant. A benefit conferred by the sheathed ErM basidiomycete to host fitness under limited nutrient supply is evidenced by the tightly coupled relationship between *Vaccinium* shoot growth and the extent of root colonization by the mycobiont as observed in the peat soil agar resynthesis medium containing no added nutrients. Associating with a ligninolytic basidiomycete differing in enzyme potential from that of ErM ascomycetes could conceivably provide the plant with a significant evolutionary advantage where lignin presents a biochemical constraint to nutrient acquisition. In addition, dual functionality as a saprotroph and mycorrhizal symbiont would widen the ecological niche of the basidiomycete, being able to persist in lignocellulose debris but shifting its carbon source to a plant in exchange for transfer of nutrients, which would allow for persistence when its initial carbon source becomes depleted to support purely saprotrophic growth. These or similar evolutionary pressures might have given rise to sheathed ericoid mycorrhiza; subsequently, carbon flow from the host plant possibly stimulated evolution of the novel lineage of basidiomycetes escaping competition with its closest relatives from Trechisporales, mostly known as soil saprobes.

### Identification of the Basidiomycete and Detection of Sheathed Ericoid Mycorrhiza

We were unable, based on the most used rDNA gene sequences, to link the basidiomycete forming sheathed ErM with any known fungal species or genus sequenced to date. Its placement into the context of recently recognized lineages of Agaricomycetes [45], [48] suggested an isolated position among orders with prevailing resupinate or polyporoid fruiting bodies and mixed saprophytic/putative mycorrhizal life style. Alternative sequence regions can further revise the basidiomycete evolutionary position as was recently shown in *Jaapia*, Strobilomycetaceae or *Wallemia* [47], [48], [63]. However, preliminary analyses of combined RNA and protein coding gene datasets (not shown) support presented nrDNA based topology. Thus, our findings unambiguously extend the range of diversity of the confirmed ErM fungi to a new fungal lineage related to, but differing from the so far described Trechisporales. Sequences of Trechisporales are infrequently detected in Ericaceae roots [77] but members of this order, or the Hymenochaetales, have never been proven as ericoid mycorrhizal fungi.

Despite the presence of sheathed ErM at significant levels in two ecosystems, the symbiosis would remain hidden when subjected to detection by ‘universal’ fungal primers utilized in the vast majority of investigations into ErM fungal diversity over the previous two decades. Similarly, Rosling *et al*. [78] documented the lack of a globally distributed, omnipresent group of Archaeorhizomycetes in the studies employing mismatching ITS primers. In addition, the basidiomycete has not been detected by isolation attempts using the basidiomycete-selective medium and randomly selected *Vaccinium* roots. These findings demonstrate that potential failures or biases inherent to a certain method may be overcome only by utilizing a combined (microscopic, culture-based and molecular) approach, potentially contributing novel, unanticipated findings to the existing knowledge of fungal ecology.

### Conclusions

The occurrence of sheathed ErM at ecologically significant levels described here may represent an overlooked mycorrhizal association of boreal Ericaceae and our findings beg further study of controls on its distribution, both locally, within and among plant individuals, and across ecosystems at the landscape and global scale. Based on our current knowledge, the basidiomycete forming sheathed ericoid mycorrhizae represents an intriguing lineage related to Trechisporales and Hymenochaetales which might have undergone rapid evolution of nrDNA genes, possibly as a consequence of a shift from free-living saprotrophic to mycorrhizal lifestyle.

## Supporting Information

Table S1 **Testing of conspecificity of the two basidiomycetes forming sheathed ericoid mycorrhiza (isolates JPK 90 =  CCF 4138 and JPK 87 =  CCF 4139) using PCR fingerprinting. DNA isolated independently from CCF 4139 was used in PCR with the following primers (see Materials and Methods):** M13-core (5′- GAGGGTGGCGGTTCT), M13 (5′-TTATGTAAAACGACGGCCAGT-3′) and 834c (5′-(AG)_8_ CG-3′) combined with 834t (5′-(AG)_8_ TG-3′). Amplifications were performed in 18.5 µl volumes, each containing 100 ng of DNA, 25 mM of MgCl2 (Promega Corp.), 0.2 mM of dNTPs and 1 U of DyNAzyme polymerase (Finnzymes), with the respective buffer. The reaction mixtures were subjected to 32 cycles under the following temperature regime: 94°C/3 min, 52°C/1 min, and 65°C/3 min (1×); 45°C/40 s, 52°C/1 min, and 65°C/3 min (35×) and 94°C/40 s, 52°C/1 min, and 65°C/10 min (1×). The amplified products were subjected to electrophoresis on 1.8% agarose gels stained with ethidium bromide, and the banding patterns were visualized under ultraviolet light. The lambda phage DNA digested with BglI rectrictase was used as a ladder.
(DOC)

Table S2 **Sequences of Basidiomycota used in the phylogenetic analyses.** We used pruned matrix from Matheny *et al*. [45], together with representatives of Amylocorticiales, Gloeophyllales and Jaapiales.
(DOC)
