# Supplementary material for: Novel Root-Fungus Symbiosis in Ericaceae: Sheathed Ericoid Mycorrhiza Formed by a Hitherto Undescribed Basidiomycete with Affinities to Trechisporales
Source: PLoS One. 2012 Jun 25;7(6):e39524. doi: 10.1371/journal.pone.0039524 (PMC3382583; doi:10.1371/journal.pone.0039524)
Supplement: Table S1 — Testing of conspecificity of the two basidiomycetes forming sheathed ericoid mycorrhiza (isolates JPK 90 = CCF 4138 and JPK 87 = CCF 4139) using PCR fingerprinting. DNA isolated independently from CCF 4139 was used in PCR with the following primers (see Materials and Methods): M13-core (5′- GAGGGTGGCGGTTCT), M13 (5′-TTATGTAAAACGACGGCCAGT-3′) and 834c (5′-(AG)8 CG-3′) combined with 834t (5′-(AG)8 TG-3′). Amplifications were performed in 18.5 µl volumes, each containing 100 ng of DNA, 25 mM of MgCl2 (Promega Corp.), 0.2 mM of dNTPs and 1 U of DyNAzyme polymerase (Finnzymes), with the respective buffer. The reaction mixtures were subjected to 32 cycles under the following temperature regime: 94°C/3 min, 52°C/1 min, and 65°C/3 min (1×); 45°C/40 s, 52°C/1 min, and 65°C/3 min (35×) and 94°C/40 s, 52°C/1 min, and 65°C/10 min (1×). The amplified products were subjected to electrophoresis on 1.8% agarose gels stained with ethidium bromide, and the banding patterns were visualized under ultraviolet light. The lambda phage DNA digested with BglI rectrictase was used as a ladder. (DOC) [file pone.0039524.s001.doc]

| 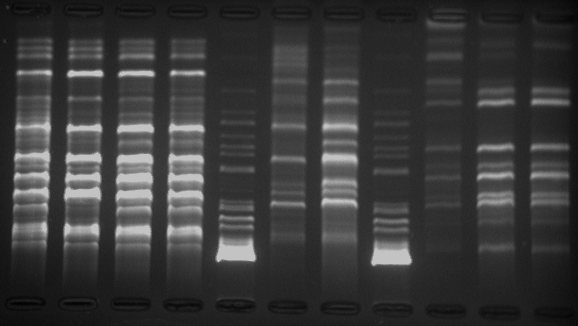 | | | | | | | | | | |
| --- | --- | --- | --- | --- | --- | --- | --- | --- | --- | --- |
| CCF 4139  DNA isolation A | CCF 4139  DNA isolation B | CCF 4139  DNA isolation C | CCF 4138 | ladder | CCF 4139 | CCF 4138 | ladder | CCF 4139  DNA isolation A | CCF 4139  DNA isolation B | CCF 4138 |
| **M13 core** | | | |  | **834c+834t** | |  | **M13** | | |
