# Supplementary material for: Novel Root-Fungus Symbiosis in Ericaceae: Sheathed Ericoid Mycorrhiza Formed by a Hitherto Undescribed Basidiomycete with Affinities to Trechisporales
Source: PLoS One. 2012 Jun 25;7(6):e39524. doi: 10.1371/journal.pone.0039524 (PMC3382583; doi:10.1371/journal.pone.0039524)
Supplement: Table S2 — Sequences of Basidiomycota used in the phylogenetic analyses. We used pruned matrix from Matheny et al. [45], together with representatives of Amylocorticiales, Gloeophyllales and Jaapiales. (DOC) [file pone.0039524.s002.doc]

| **Species and taxonomical placement** | **18S**  **Accession** | **25S**  **Accession** | **5.8S (ITS)**  **Accession** |
| --- | --- | --- | --- |
| **Agaricomycetes sp. CCF 4138** | HE573028 | | |
| **Agaricales** |  |  |  |
| *Agaricus bisporus* | AY787216 | AY635775 | DQ404388 |
| *Agrocybe praecox* | AY705956 | AY646101 | AY818348 |
| *Ampulloclitocybe clavipes* | AY771612 | AY639881 | AY789080 |
| *Anthracophyllum archeri* | DQ092915 | AY745709 | DQ404387 |
| *Armillaria mellea* | AY787217 | AY700194 | AY789081 |
| *Asterophora lycoperdoides* | DQ367417 | AF223190 | AF357037 |
| *Bolbitius vitellinus* | AY705955 | AY691807 | DQ200920 |
| *Calocybe carnea* | DQ367418 | AF223178 | AF357028 |
| *Cantharocybe gruberi* | DQ234546 DQ234547 | DQ234540 | DQ200927 |
| *Chlorophyllum agaricoides* | AY657010 | AY700187 | DQ200928 |
| *Chondrostereum purpureum* | AF082851 | AF518607 | DQ200929 |
| *Clavaria zollingeri* | AY657008 | AY639882 | AY854071 |
| *Clavulinopsis laeticolor* | DQ437680 | AY745693 | DQ202267 |
| *Clitocybe candicans* | AY771609 | AY645055 | DQ202268 |
| *Collybia tuberosa* | AY771606 | AY639884 | AY854072 |
| *Coprinopsis cinerea* | Genome | AF041494 | AF345819 |
| *Coprinus comatus* | AY665772 | AY635772 | AY854066 |
| *Cortinarius aurilicis* | AY705957 | AY684152 | DQ083772 |
| *Crepidotus* cf. *applanatus* | AY705951 | AY380406 | DQ202273 |
| *Cylindrobasidium laeve* | AF518576 | DQ234541 | AY536283 |
| *Entoloma prunuloides* | AY665784 | AY700180 | DQ206983 |
| *Flammulina velutipes* | AY665781 | AY639883 | AY854073 |
| *Henningsomyces candidus* | AF334916 | AF287864 | AY571043 |
| *Hygrocybe* aff. *conica* | AY752965 | AY684167 | AY854074 |
| *Inocybe dulcamara* | AY657016 | AY700196 | DQ221106 |
| *Lepista irina* | AY705948 | DQ234538 | DQ221109 |
| *Lycoperdon pyriforme* | AF026619 | AF287873 | AY854075 |
| *Lyophyllum leucophaeatum* | DQ367420 | AF223202 | AF357032 |
| *Mythicomyces corneipes* | DQ092917 | AY745707 | DQ404393 |
| *Nia vibrissa* | AF334754 | AF334750 | AY571053 |
| *Nolanea sericea* | DQ367421 | DQ367423 | DQ367430 |
| *Phaeomarasmius proximans* | AY752970 | AY380410 | DQ404381 |
| *Phyllotopsis* sp. | AY707090 | AY684161 | DQ404382 |
| *Pluteus romellii* | AY657014 | AY634279 | AY854065 |
| *Rhodocollybia maculata* | AY752966 | AY639880 | DQ404383 |
| *Schizophyllum commune* | X54865 | AF334751 | AF249390 |
| *Tricholoma myomyces* | DQ367422 | U76459 | AF319428 |
| *Tricholomopsis decora* | DQ092914 | AY691888 | DQ404384 |
| *Tubaria confragosa* | AY665776 | AY700190 | DQ267126 |
| *Xerula radicata* | AY654884 | AY645051 | DQ241780 |
| **Amylocorticiales** |  |  |  |
| *Amylocorticium subsulphureum* | GU187617 | GU187562 | GU187506 |
| **Atheliales** |  |  |  |
| *Fibulorhizoctonia* sp. | AY654887 | AY635779 | AY854062 |
| **Auriculariales** | AY654887 | AY635779 | AY854062 |
| *Auricularia* sp. | DQ234542 | AY634277 | DQ200918 |
| *Bourdotia* sp. | DQ234544 DQ234545 | AY635777 | DQ200925 |
| *Exidia glandulosa* | AY293129 | AY293179 | AY509555 |
| **Boletales** |  |  |  |
| *Aureoboletus thibetanus* | AY654882 | AY700189 | DQ200917 |
| *Boletellus projectellus* | AY662660 | AY684158 | AY789082 |
| *Boletinellus merulioides* | AY662668 | AY684153 | DQ200922 |
| *Calostoma cinnabarinum* | AY665773 | AY645054 | AY854064 |
| *Coniophora arida* | AY293123 | AF098375 | DQ202271 |
| *Hygrophoropsis aurantiaca* | AY662663 | AY684156 | AY854067 |
| *Paxillus vernalis* | AY662662 | AY645059 | DQ267128 |
| *Serpula himantioides* | AF518589 | AF518648 | AJ536025 |
| *Strobilomyces floccopus* | AY662661 | AY684155 | AY854068 |
| *Suillus pictus* | AY662659 | AY684154 | AY854069 |
| **Cantharellales** |  |  |  |
| *Botryobasidium subcoronatum* | AY662666 | AY647212 | DQ200924 |
| *Clavulina* sp. | AY757265 | AY745694 | DQ202266 |
| *Hydnum albomagnum* | AY665777 | AY700199 | DQ218305 |
| *Sistotrema confluens* | AY757260 | AY647214 | DQ267125 |
| *Sistotrema coronilla* | AY757259 | DQ457641 | DQ397337 |
| **Corticiales** |  |  |  |
| *Punctularia strigosozonata* | AF518586 | AF518642 | DQ398958 |
| *Vuillemenia comedens* | AF518594 | AF518666 | DQ398959 |
| **Dacrymycetes** |  |  |  |
| *Calocera cornea* | AY771610 | AY701526 | AY789083 |
| *Guepiniopsis buccina* | - | AY745711 | DQ206986 |
| **Gloeophyllales** |  |  |  |
| *Veluticeps abietina* | HM536080 | HM536079 | - |
| **Jaapiales** |  |  |  |
| *Jaapia argillacea* | GU187524 | GU187581 | AF518581 |
| **Phallomycetidae** |  |  |  |
| *Gautieria otthii* | AF393043 | AF393058 | AF377072 |
| *Phallus hadriani* | AY771601 | AY885165 | DQ404385 |
| *Ramaria rubella* | AY707095 | AY645057 | AY854078 |
| **Hymenochaetales** |  |  |  |
| *Coltricia perennis* | AF026583 | AF287854 | DQ234559 DQ234560 DQ234561 |
| *Cotylidia* sp. | AY705958 | AY629317 | AY854079 |
| *Fomitoporia mediterranea* | AY662664 | AY684157 | AY854080 |
| *Hydnochaete duportii* | AY662669 | AY635770 | DQ404386 |
| *Hyphoderma praetermissum* | AY707094 | AY700185 | AY854081 |
| *Resinicium bicolor* | AF026615 | AF393061 | DQ218310 |
| *Rickenella fibula* | AY771599 | AY700195 | DQ241782 |
| **Polyporales** |  |  |  |
| *Albatrellus higanensis* ined. | AY707091 | AY684166 | AY789078 |
| *Climacodon septentrionalis* | AY705964 | AY684165 | AY854082 |
| *Fomitopsis pinicola* | AY705967 | AY684164 | AY854083 |
| *Ganoderma tsugae* | AY705969 | AY684163 | DQ206985 |
| *Grifola frondosa* | AY705960 | AY629318 | AY854084 |
| *Laetiporus sulphureus* | AY705966 | AY684162 | DQ221108 |
| *Phaeolus schweintizii* | AY705961 | AY629320 | AY218422 |
| *Phanerochaete chrysosporium* | AF026593 | AF287883 | AY854086 |
| *Phlebia radiata* | AF026606 | AF287885 | AY854087 |
| *Polyporus arcularius* | AF334928 | AF393067 | AF516523 |
| *Polyporus squamosus* | AY705963 | AY629320 | DQ267123 |
| *Pycnoporus* sp. | AY705970 | AY684160 | DQ411525 |
| *Sparassis crispa* | AY705962 | AY629321 | DQ250597 |
| *Spongipellis pachyodon* | DQ457638 | AY629322 | DQ249277 |
| *Trametes versicolor* | AY706965 | AY684159 | AY354226 |
| **Russulales** |  |  |  |
| *Bondarzewia montana* | U59063 | DQ234539 | DQ200923 |
| *Echinodontium tinctorium* | AF026578 | AF393056 | AY854088 |
| *Hericium americanum* | AY665778 | DQ411538 | DQ206987 |
| *Heterobasidion annosum* | AF026576 | AF287866 | DQ206988 |
| *Lactarius deceptivus* | AY707093 | AY631899 | AY854089 |
| *Peniophora nuda* | AF026586 | AF287880 | DQ411533 |
| *Sterum hirsutum* | AF026588 | AF393078 | AY854063 |
| **Sebacinalles** |  |  |  |
| *Tremellodendron pallidum* | AY766081 | AY745701 | DQ411526 |
| **Thelephorales** |  |  |  |
| *Hydnellum geogenium* | AY752971 | AY631900 | DQ218304 |
| *Polyozellus multiplex* | AY771600 | AY634275 | DQ411528 |
| **Trechisporales** |  |  |  |
| *Trechispora alnicola* | AY657012 | AY635768 | DQ411529 |
| *Trechispora* sp. | AY803753 | AY647217 | DQ411534 |
| **Tremellomycetes** |  |  |  |
| *Cryptococcus neoformans* | X60183 | AJ551290 | AF444326 |
